# Impact and challenges of enactment for advanced regenerative medicine in South Korea

**DOI:** 10.3389/fbioe.2022.972865

**Published:** 2022-10-12

**Authors:** Dong-Sook Kim, SeungJin Bae

**Affiliations:** ^1^ Department of Research, Health Insurance Review and Assessment Service, Wonju, South Korea; ^2^ College of Pharmacy, Ewha Womans University, Seoul, South Korea

**Keywords:** regenerative medicine, enactment, biological products, safety, South Korea

## Abstract

The Korean government has enacted the Act on Advanced Regenerative Medicine and Advanced Biological products (ARMAB) in August 2019, and it has been implemented in 2020. We reviewed the changes made by ARMAB compared to the existing Pharmaceutical Affairs Act and discussed future challenges to accelerate regenerative medicine while ensuring safety and efficacy. This act and regulations focused on the key elements of act as follows: the definition of advanced regenerative medicine (RM), the licensing of related facilities, safety management such as long-term follow-up, clinical research review committee, and establishment of a roadmap. Our study shows that Korea has achieved the second highest number of first approvals for regenerative medicine indications worldwide through expedited approvals encouraging innovation, while maintaining patient safety by mandating long-term follow-up. Additionally, the establishment of an interactive system for retrieval of patients' data and reporting of safety information by manufacturers electronically demonstrates Korea’s commitment to innovation for Advanced RM and patient safety.

## Introduction

With the increase of geriatric syndromes and rare incurable diseases due to aging, the demand for regenerative medicine (RM) is increasing (Cossu et al., 2018). RM or cell therapy products (CTP) have the potential to repair or reconstruct damaged functional cells and tissues for unmet medical needs such as dementia and organ defects (Witten et al., 2015).

Since RM uses live cells and tissues to repair or reconstruct damaged cells and tissues, preventing microbial contamination and maintaining living cells require other processes different from chemical medicines in terms of manufacturing procedures such as sterilization and aseptic testing, validation, collection of the cells, cultivation, production, and follow-up (Giancola et al., 2012). Thus, this process is the key driver for raising the manufacturing cost of RM. In addition, since RM involves personalized treatment or rare diseases, and thus, there are only a few patients targeted for RMs, it is difficult to secure a sufficient number of patients in clinical trials for regulatory approval (Abou-El-Enein et al., 2016). To compensate for this limitation, post-marketing surveillance for tracking long-term side effects is needed (Hara et al., 2014). Therefore, RM requires a different authorization framework from the current licensing system.

Several countries and the European Union (EU) have established specific legislation for RMs and adopted special requirements for achieving regulatory exemption of RM approval in the EU, the United States, Japan, and Australia (European Medicines Agency, 2007; Azuma, 2015; Food and Drug Administration, 2016; Grounds, 2018; Qiu et al., 2020). In the United States, “the 21st Century Cures Act” contains new legislation affecting cell therapy in 2016, such as the Regenerative Medicine Advanced Therapy (RMAT) designation and “Expedited Programs for RM Therapies for Serious Conditions,” including fast track designation, accelerated approval, and priority review designation (Food and Drug Administration, 2016; Kang et al., 2021).

The EU introduced regulation for Advanced Therapy Medicinal Products (ATMPs) in 2007 (European Medicines Agency, 2007). According to Article 28 [the European Commission (EC) regulation of No 1394/2007], each EU member state can apply conditions for the exemption of products from the centralized marketing authorization for ATMPs [referred to as hospital exemption (HE)]. These include the application of specific quality standards, use under the exclusive professional responsibility of medical practitioner and the national traceability, and pharmacovigilance, *etc.* (Cuende et al., 2022).

The Japanese government enacted a suite of laws and amendments specifically designed for the regulation of RM. In 2014, Japan enacted the Pharmaceutical and Medical Device Act (PMD Act) to include a unique regulatory conditional, time-limited market access pathway for RM (Hara et al., 2014; Azuma, 2015; Maeda et al., 2015), and then the Act on the Safety of Regenerative Medicine (RM Act) to establish a framework for RM and clarify measures necessary for ensuring patient safety (Azuma 2015; Tobita et al., 2016).

Globally, the number of companies producing RMs has increased from 772 in 2016 to 1,085 in 2020, and the size of the international RM market in 2020 is calculated to be 27.29 billion USD (Research, 2021). In Korea, the manufacturing of cell therapy has grown at an average annual rate of 29.7%, from 18 million USD in 2014 to 53.57 million USD in 2018, but the number of approved medications in Korea has shown a slowdown since 2015 (Kim et al., 2021). Moreover, the number of clinical trials for RM in Korea increased from 15 in 2010 to 27 in 2012, but the number of clinical trials has been stagnant since 2017.

In Korea, to accelerate R&D on RM as well as CTP, the discussion has been ongoing since 2013, and the Act on the safety of and support for Advanced Regenerative Medicine and Advanced Biological products (ARMAB) was enacted in August 2019 and has been executed since 2020 (Ministry of Health and Welfare, 2020a). This study aimed to describe the history of the act and the details of ARMAB in South Korea, and thereby suggest the landscape for future challenges.

## Backgrounds of legislation

First, the legislation was introduced to establish a legal basis for RMs, which were processed with minimal operation/processing. At the time of 2019, the Pharmaceutical Affairs Act only specified the method of approval of CTP manufactured by pharmaceutical companies, and there was no legal basis for collecting and administering somatic cells or adult stem cells to patients in medical facilities with minimal operations. Since somatic cells or adult stem cells were not included in the extent of CTP in the Pharmaceutical Affairs Act, the enactment of the new law has provided the legal basis to enable medical facilities to culture cells and process for RM. Before the legislation, the RMs was not defined as “use a legitimate medical service”. In Korea, the payment system for both inpatient and outpatient care is based on a fee-for-service, which defines all of the covered and uncovered medical services in the health insurance benefits scheme. Second, the new law covered RM as well as advanced biopharmaceuticals and integrated all related regulations into one new Act. In the past, regulations related to biopharmaceuticals were scattered in the Pharmaceutical Affairs Act, the Bioethics Act, and the Blood Management Act, but now, they have been unified through the ARMAB Act. The third purpose of enacting the law was for the government to consider biopharmaceuticals as a new industry for future growth and to establish a basis for supporting them. The new act has allowed patients who had no available treatment to receive RM as part of clinical research for RM to be enabled in patients who have no traditional treatment. Nonetheless, it provided the basis for supporting R&D and data governance for RM as well as advanced biopharmaceuticals. By enacting the law, the government should establish a roadmap and action plan, and thereby create the budget for funding of R&D. Based on this law, the government can designate RM support institutions and advanced biopharmaceutical regulatory science centers. Lastly, the new act introduced both the expedited approval and review method and post-market risk management. To strengthen the safety of advanced biopharmaceuticals, a safety management procedure for the entire life cycle from cell collection to final use has been prepared. Long-term follow-up management is legally obligatory.

However, the process of enactment took a long time. The preparation process for the Act started in 2013, after which, several bills were proposed from 2016 to 2018, and opinions from relevant government ministries and the private sector were collected. The final bill was introduced in March 2019, passed by the Legislative Judiciary Committee and the National Assembly plenary session on 31 July 2019, and 2 August 2019, respectively.

After the legislation was passed in 2019, as shown in Table 1, the enforcement ordinance and administrative rules were enacted in 2020 (Ministry of Food and Drug Safety, 2019; Ministry of Food and Drug Safety, 2020a; Ministry of Health and Welfare, 2020a; Ministry of Food and Drug Safety, 2020b; Ministry of Health and Welfare, 2020b; Ministry of Food and Drug Safety, 2020c; Ministry of Health and Welfare, 2020c; Ministry of Food and Drug Safety, 2020d; Ministry of Health and Welfare, 2020d; Ministry of Health and Welfare, 2021). The subordinate statutes included the expedited approval procedure and plan for safety management of advanced biopharmaceuticals reflecting the specificity of RM and advanced biopharmaceuticals. In addition, it includes the establishment of a management system to review and support clinical research on RM at the national level and a strategy to promote clinical research on RM.

**TABLE 1:** Regulations on cellular treatment in South Korea.

Category	Act and regulation	Year
Acts	- Act on the safety of and support for Advanced Regenerative Medicine and Advanced Biological Products (Ministry of Health and Welfare, 2020a)	2019
	- Enforcement decree on the safety of and support for Advanced Regenerative Medicine and Advanced Biological Products (Ministry of Health and Welfare, 2021)	
Regulationss	- Regulations on approval and safety of human cells, etc. and Advanced Biopharmaceuticals (Ministry of Food and Drug Safety, 2020a)	2020
	- Regulations on the recall and disposal order of hazardous Human Cells, etc*.* (Ministry of Food and Drug Safety, 2020b)	
	- Rules on safety and support of Advanced Biopharmaceuticals (Ministry of Food and Drug Safety, 2020c)	
	- Regulations on the designation of Advanced RM implementing institution and matters to be observed in the cell processing business (Ministry of Health and Welfare, 2020b)	
	- Regulations on the preparation, submission, and review of Advanced Regenerative Medicine research plans, etc. (Ministry of Health and Welfare, 2020c)	
Guideline etc.	- Management Standard for Long-term Follow-up of Advanced Biopharmaceuticals (Ministry of Health and Welfare, 2020d)	2021
	- Guidelines for evaluating the data integrity of biopharmaceutical manufacturers (Ministry of Food and Drug Safety, 2020d)	

The significant change is to divide past RM into regenerative medical treatment and biopharmaceutical medicinal products. Thus, it differs from the regulation in European Union or other countries such as United States and Australia, which involves medicinal products referred to as ATMPs or RMAT in that this law covers both RMs and advanced biopharmaceuticals. This is because, in Korea, all medical services provided by medical institutions, except for cosmetic and cosmetic purposes, must be defined as covered or non-covered in the health insurance benefits list. Also, this law includes long-term follow-up requirements for patient safety.

**FIGURE 1:**
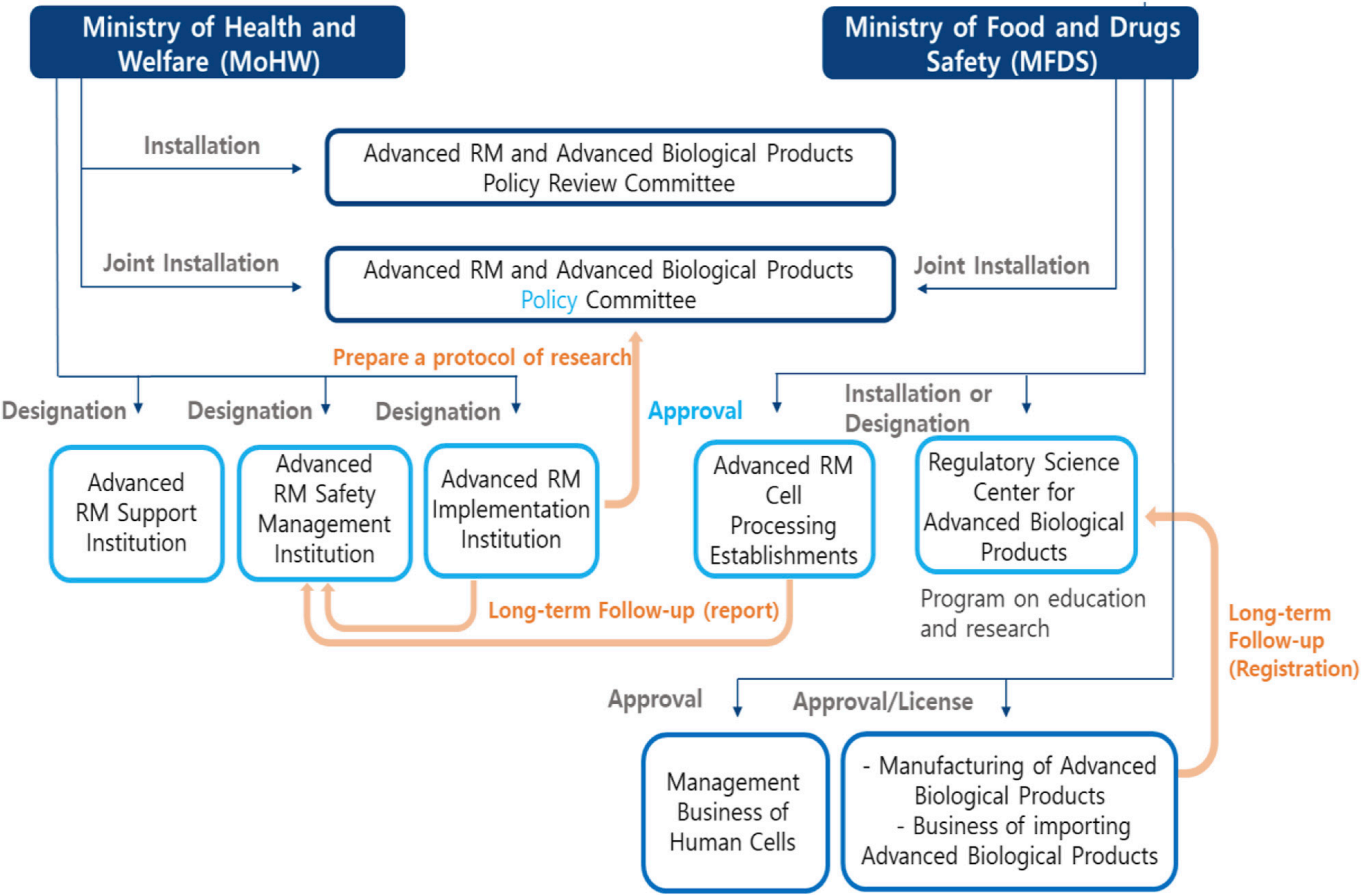
Relevant institutions of Advanced RM and Advanced biopharmaceuticals.

The role and relationship between each institution is shown in Figure 1. The structure of this law is shown in Table 2.

**TABLE 2:** The structure of the ARMAB Act (Ministry of Health and Welfare, 2020a).

Advanced RM	Advanced biopharmaceuticals
- Definition and scope (Article 2 No. 1)	- Definition and scope (Article 2 No. 5)
- Establishment and designation of Advanced RM related Support Agency (Article 9)	- Licensing of pharmaceutical manufacturers (Articles 23, 24, and 25) and import business (Article 27)
- Designation of Advanced RM institution (Article 10)	- The obligations and reporting on the quality control of manufacturing (Article 26)
- Consent for clinical research (Article 11)	- Licensing for the management of human cells (Articles 28 and 29)
- Review committee on the proposal of clinical research (Articles 13 and 14)	- Safety monitoring and Long-term follow-up (Article 30)
- The facility of cell processing on Advanced RM (Articles 15, 16, 17, and 18)	- Establishment and operation of Regulatory Science Center (Articles 32, 33, and 34)
- Safety management agency of Advanced RM (Article 19)	
- Safety monitoring and adverse event reporting (Article 20)	- The designation of products that apply for the expedited approval review (Article 36)
- Long-term follow-up of clinical research patients (Article 21)	

## The key elements of advanced regenerative medicine and advanced biological products act

As shown in Table 3, we classified the key elements of the new act into five categories as follows: 1) terminology of Advanced RM and classification of clinical research, 2) establishment of roadmap and operation of Policy Committee, 3) the regulation on relevant institutions of Advanced RMs (Advanced RM-related Support Agency, Advanced RM institution, clinical research proposal Review Committee of Advanced RM, the facility of cell processing, and safety management agency), 4) the regulation on relevant institutions of Advanced biopharmaceuticals (licensing for the Advanced biopharmaceutical manufacturing or import business, licensing for the collection and supply of human cells, and establishment and operation of Regulatory Science Center), and 5) long-term follow-up and safety management.

**TABLE 3:** Key elements of ARMAB Act: Definition, the criteria of the facility (MFDS, 2020a; Ministry of Health and Welfare, 2020a; Ministry of Health and Welfare, 2020b; Ministry of Health and Welfare, 2020c; Ministry of Health and Welfare, 2021)

	Advanced RM	Advanced biopharmaceuticals
Definition and scope	Advanced RM is defined as therapies that process human cells that are intended to be used for either the regeneration, repair, and formation of human body structures or functions or the treatment or prevention of human diseases, which includes cell therapy products (CTP), gene therapy products (GTP), and tissue-engineered products (TEP)	Advanced biopharmaceuticals includes cell therapy products (CTP), gene therapy products (GTP), tissue-engineered products (TEP), and advanced bio-convergence products
	Human cells include stem cells, hematopoietic stem cells, somatic cells, immune cells, and xenogeneic cells derived from the human body	
Player	Medical institutions	Pharmaceutical companies
Type of research	Clinical research in advanced RM is divided into high-risk, medium-risk, and low-risk groups by examining the impact on human life and health and the degree of risk. Clinical research on advanced RM can be conducted after approval by a Review Committee	A clinical trial is according to existing Pharmaceutical Law
Establishment and implementation of the roadmap	The government should establish a basic roadmap every 5 years, including improving regulation, supporting R&D, commercialization, patient safety management, and financing	
	The Policy Committee should be operated by the MoHW, and it consists of 21 members	
Relevant institutions	- Advanced RM related Support Agency (Designation by the Ministry of Health and Welfare (MoHW))	- The obligation of manufacturing and import, including criteria of quality control
	- Advanced RM institution (Designation by the MoHW)	- Licensing for the collection and supply of human cells (approval by the MFDS)
	- Review Committee on the proposal of clinical research (Operation by the Ministry of Health and Welfare, and MFDS)	- Establishment and operation of a Regulatory Science Center (Designation by the MFDS)
	- The facility of cell processing on Advanced RM (Approval by the Ministry of Food and Drug Safety (MFDS))	
	- Safety management agency of Advanced RM (Designation by the MoHW)	
Follow-up and safety management	A long-term follow-up investigation for clinical research has been decided by the Review Committee and a long-term follow-up is necessary	In order to strengthen the safety management of the administered patients, targets for “long-term follow-up management” are designated, and serious adverse events are followed up for a certain period or long term after administration

### Definition and classification of research

Advanced RM is defined as therapies that process human cells that are intended to be used for either the regeneration, repair, and formation of human body structures or functions or the treatment or prevention of human diseases, which include CTP, gene therapy products (GTP), and tissue-engineered products (TEP), whereas Advanced biopharmaceuticals includes advanced bio-convergence products, additionally. Human cells include stem cells, hematopoietic stem cells, somatic cells, immune cells, and xenogeneic cells derived from the human body.

A review committee of a clinical research proposal is established to encourage clinical research for patients without alternative treatment. Clinical research in advanced RM is divided into high-risk, medium-risk, and low-risk groups by examining the impact on human life and health and the degree of risk. Clinical research on advanced RM can be conducted after approval by a review committee jointly operated by the Ministry of Health and Welfare (MoHW) and the Ministry of Food and Drug Safety (MFDS).

### Roadmap establishment and governance

The ARMAB stipulated that the government should establish a basic plan every 5 years, including improving regulation, supporting R&D, commercialization, patient safety management, and financing.

To establish and implement the roadmap, the governance is operated by the MoHW, the MFDS, and the Policy Review Committee operated by the MoHW, which consists of 21 members.

Moreover, this act specified relevant government institutions and agencies of regulations. First, Ministry of Health and Welfare, might establish or designate an Advanced RM-related Support Agency according to Article 9. This agency should conduct research, human resource training, the support of industrial infrastructure, and international cooperation in the field of RMs. Second, according to Article 10, the Advanced RM implementation institution should get the designation of Ministry of Health and Welfare, and be responsible for clinical research with human cells supplied from cell processing facilities. The Advanced RM implementation institution should get the informed consent of the subjects participating in the research and apply for a review of the clinical research proposal, and then obtain approval. Third, the facility of cell processing on Advanced RM should get the approval of MFDS according to Article 15. Fourth, in order to ensure safety, Ministry of Health and Welfare, should designate the safety management agency of Advanced RM among affiliated government institutions of Ministry of Health and Welfare, according to Article 19. The designated agency must carry out safety monitoring and long-term follow-up of clinical research.

### Licensing of pharmaceutical companies and management business

In order to produce and import Advanced biopharmaceuticals, manufacturers, and import businesses should get licensed by MFDS. After obtaining approval, the manufacturing and management obligations should comply with the standards of each step, including facility, equipment, manpower, and quality control of the process from the cell collection stage to the inspection, processing, storage, *etc.*, according to Articles 25, 26, and 27. Nonetheless, the business that collects and supplies human cells should obtain licensing by MFDS according to Articles 28 and 29. In addition, this act includes the establishment and operation of the Regulatory Science Center by MFDS according to Article 32. This agency should conduct research, human resource training, support industrial infrastructure, and international cooperation.

### Safety management

The main challenge of the enactment is to ensure safety and protect vulnerable patients from the risk of uncertainty about the evidence of new therapies. For the Advanced RM, the clinical research proposal Review Committee of Advanced RM should review and decide whether the proposal of research is appropriate according to Articles 12 and 14. The safety management agency of Advanced RM should conduct a long-term follow-up for clinical research that the Review Committee recommended a long-term follow-up according to Article 21.

For Advanced biopharmaceuticals, in order to strengthen the safety management, targets for “long-term follow-up management” are designated by MFDS, and serious adverse events are followed up for a certain period or long term after administration according to Article 30. Companies that have obtained approval for clinical trial protocols or medicine products designated as long-term tracking targets must report drug sales and supply details into the Regulatory Science Center system, and any adverse reactions to the MFDS.

### The expedited approval review of advanced biopharmaceuticals

According to Article 36, for the designation of the target for fast review, the head of MFDS may designate the applied cell therapies as the subject of “fast review” under certain conditions, such as (1) where there is no alternative treatment and the purpose is to treat serious life-threatening diseases, such as cancer; (2) where the purpose is to treat rare diseases under the Rare Disease Control Act; and (3) where the purpose is to prevent or treat the pandemic of bioterrorist infectious diseases and other infectious diseases under the Act on the Prevention and Management of Infectious Diseases (Fujita and Kawamoto, 2016).

For advanced biopharmaceuticals, commercialization is supported by customized review, priority review, or conditional approval. Each program is as follows: 1) customized review: step-by-step pre-screening by submitting permission data in advance according to the developer’s schedule, 2) priority review: priority review over other drugs, and 3) conditional approval: drugs used for serious and rare diseases are approved as data from phase 2 clinical trials under the condition that phase 3 clinical trials will be conducted after marketing. The expedited approval might be expected to reduce the time required for new drug approval by 3.5–4.5 years.

### Changes of advanced biopharmaceuticals

In addition, as this law was enacted, changes were made as shown in Table 4. This change was focused on Advanced biopharmaceuticals.

**TABLE 4:** Changes of advanced biopharmaceutical related regulation before and after ARMAB Act (MFDS, 2019; Ministry of Food and Drug Safety, 2020a; Ministry of Health and Welfare, 2020a).

	Past (pharmaceutical Act)	After the law ARMAB
Aim	The aims were to secure the safety and efficacy of cell therapy products in the application for approval	The aims were to integrate related regulations into one new act and to establish a legal basis for RM. Moreover, the new act introduced both the expedited approval and review method and post-market risk management
		By enacting the law, the government should establish a roadmap and action plan
Definition	Biopharmaceuticals includes biologics, cell therapy products (CTP), gene therapy products (GTP), tissue-engineered products (TEP)*etc.*	Advanced biopharmaceuticals are CTP, GTP, TEP, and advanced bio-convergence products
	However, cases where doctors perform only minimal manipulations such as simple separation, washing, freezing, and thawing of autologous or allogenic cells during treatment are excluded	However, cases where doctors perform only minimal manipulations such as simple separation, washing, freezing, and thawing of autologous or allogenic cells during treatment are excluded
The collection and supply of cell	A pharmaceutical manufacturer collects, processes, stores, and uses living cells by maintaining their specific properties, and the collection is conducted by medical institutions	A company that collects, imports, inspects, processes, and supplies human cells as raw materials for Advanced Biopharmaceuticals should obtain a license for the management of human cells*etc.*
		The requirement of ethics is added to the pharmaceutical manufacturers as follows: cell and tissue donor suitability assessments are mandatory
		(A facility of cell processing on Advanced RMs must obtain a license.)
The quality control of manufacturing	Cell therapy products are managed according to GMP standards in accordance with the same regulations as other pharmaceuticals	From the cell collection stage to the inspection, processing, storage, etc., it is necessary to manage each step of the process. In addition, the provision of a “record management room” has been made compulsory for manufacturing/import permits
Follow-up	A long-term follow-up investigation must be conducted in accordance with the regulations, and a fine of not more than 1.8 thousand USD Won is imposed in the case of violation	In order to strengthen the safety management of the administered patients, targets for “long-term follow-up management” are designated and serious adverse events are followed up for a certain period or long term after administration. Violations are punishable by up to 5 years in prison or a fine of up to 45,000 USD.
The expedited approval review	The expedited approval review program was operated in accordance with the announcement and guidelines	The legal basis for the expedited approval review program has been clarified
		- Customized review: step-by-step pre-screening by submitting permission data in advance according to the developer’s schedule
		- Priority review: priority review over other drugs
		- Conditional approval: drugs used for serious and rare diseases are approved as data from phase 2 clinical trials under the condition that phase 3 clinical trials will be conducted after marketing

From the cell collection stage to the inspection, processing, storage, *etc.*, it is necessary to manage each step of the process. In addition, the provision of a “record management room” has been made compulsory for manufacturing and import permits. Moreover, a company that collects, imports, inspects, processes, and supplies human cells as raw materials for Advanced Biopharmaceuticals should obtain a license for the management of human cells, and cell and tissue donor suitability assessments are mandatory.

## The advanced regenerative medicine and advanced biological’s challenge

### Achievements

Regarding RM, the Regenerative Medicine Acceleration Foundation in Korea has been designated as the advanced RM support institution, and 34 institutions have been designated as the Advanced RM implementation institution, as of December 2021, which has been increased to 43 institutions in May 2022. Twenty seven medical research proposals for the RM were applied, and five of them were approved as of 1 June 2022. Systems related to the safety of RM have been gradually installed in the Korea Disease Control and Prevention Agency and the Korean National Institute of Health and are scheduled to open until 2022. Additionally, 19 cell treatment institutions have been designated so far.

The review system for advanced biopharmaceuticals has been reorganized. Since the enactment of the Act one advanced biopharmaceutical has been approved, which is Kymriah^®^. 52 biopharmaceuticals had been approved as of December 2021, where 18 of them were advanced biopharmaceuticals (15 CTP, three GTP), and all of them obtained re-approval from the regulatory body. The MFDS has clarified the process for the priority review, and reduced review period from 115 to 90 days. Furthermore, to enhance transparency and consistency of the review, the review results are open to the public within 180 days of the review, and the opinions from the industries are sought within 30 days of the review.

The Korea Institute of Drug Safety & Risk Management (KIDS) has been designated as the Regulatory Science Center for advanced biopharmaceuticals. To lay the foundation for guaranteeing the quality and safety of the post-marketing products, long-term follow-up systems (research plan, sales, and supply) have been planned, and systems for evaluating the quality of the products are being prepared step-by-step.

Also, based on the ARMAB, long-term, electronic follow-up of advanced biopharmaceuticals is established as of November 2021, which allowed patient-centered safety management by letting health care providers and manufacturers to report electronically and retrieve patients’ information who had received the advanced biopharmaceuticals.

### Challenges to encourage innovation

To activate the conduction of the clinical trials, financial support, providing proper infrastructure should be planned ahead. First, the Korean Government has assigned 30 million USD for this project, and it has implemented various activities such as constructing GMP facilities and empowerment of the hospitals’ cell management capacities. Yet, the deficiencies of the experts capable of conducting research or regulatory affairs are emphasized. Second, the lack of manufacturing infrastructure capable of cell research could be a barrier for small-sized companies. To overcome these problems, the Korean Government has announced the plan for the “Advanced biopharmaceuitlcas-2025” and provided schemes such as joint research and outsourcing manufacturing. Third, after the RMs are approved and produced as commercial products, the reimbursement of the RM is a challenge, because their prices are extremely high since their population size is very limited. If the RM is used as non-listed drugs, then the widespread use of the RMs could be difficult. Finally, patient safety, especially among the vulnerable population, is critical. Various measures to evaluate the safety of the RMs should be followed.

Despite those limitations, in order to maximize the potential value of the RM, an infrastructure for encouraging RM innovation and sharing experience on a global scale is required.

### Proposals for foreign countries

According to Qiu et al. (2020), a total of 55 RMs were granted market access (MA) and the number of first approval for regenerative medicine worldwide was highest in the United States, followed by Korea, EU, Japan, and India (Qiu, Hanna et al., 2020). As of 2019, 16 RMs were approved in Korea, thus an accelerated approval program was needed for rapid market access. In other countries, whereas European Union (EU) and Japan enacted specific legislation for RM, the United States comprehensive act contains affecting cell therapy. In accordance with the these act, RMs got accelerated regulatory programs as follows: Regenerative Medicine Advanced Therapy (RMAT) designations in the US by the Food and Drug Administration (FDA), Priority medicine (PRIME) designation by the European Medicines Agency (EMA), and SAKIGAKE (fore-runner initiative) designation by the Pharmaceuticals and Medical Devices Agency (PMDA) in Japan. In addition to the accelerated approval program, the need for financial support was raised to promote research and development. Therefore, Korea has implemented an Act on the safety of and support for ARMAB, which has differences from other countries.

In conclusion, our study provides specific details of the ARMAB act, and we believe the Korean case could be a nice example for other countries which sought to enhance R&D for ARMAB as well as protect patient safety because this law includes long-term follow-up requirements for patient safety. The ARMAB is the first, interactive system in the world, which allowed patients’ follow-up information to be retrieved while letting manufacturers report safety information electronically. Also, since the regulation of RM and Advanced biological products is important for the field, related academic society should develop and suggest internationally acceptable guideline including definition and standard of RM. At the same time, further study should be conducted to monitor its impact on patient safety and the innovation of the ARMAB.
